# Prokineticin 2/PROK2 and Male Infertility

**DOI:** 10.3390/biomedicines10102389

**Published:** 2022-09-25

**Authors:** Carla Petrella, Matteo Spaziani, Valerio D’Orazi, Luigi Tarani, Sergio Terracina, Francesca Tarani, Ginevra Micangeli, Christian Barbato, Antonio Minni, Antonio Greco, Andrea M. Isidori, Giampiero Ferraguti, Marco Fiore

**Affiliations:** 1 Institute of Biochemistry and Cell Biology, Section of Neurobiology, National Research Council (IBBC-CNR), 00161 Rome, Italy; 2 Department of Experimental Medicine, Section of Medical Pathophysiology, Food Science and Endocrinology, Sapienza University of Rome, 00161 Rome, Italy; 3 Department of Surgical Sciences, Sapienza University of Rome, 00161 Rome, Italy; 4 Department of Pediatrics, Sapienza University of Rome, 00161 Rome, Italy; 5 Department of Experimental Medicine, Sapienza University Hospital of Rome, 00161 Rome, Italy; 6 Department of Sense Organs, Sapienza University Hospital of Rome, 00161 Rome, Italy

**Keywords:** male infertility, PROK system, PROK2, pre-clinical and clinical study, azoospermia, biomarker

## Abstract

Male infertility represents about 50% of the causes of infertility in couples. The diagnosis process represents an important procedure for defining, when possible, the causes and approaching treatments (pharmacological, surgical) aimed at overcoming the problem. Several scientific studies have set out to discover early and indicative markers capable of providing information on the biological origin of infertility and increase current knowledge in the context of new potential therapeutic approaches. The prokineticin system (PROK) consists of the prokineticin 1 (PROK1) and prokineticin 2 (PROK2) proteins. Through the activation of two G-protein receptors (PROKR1 and PROKR2) regulate a wide range of biological functions, including gastrointestinal motility, circadian rhythm regulation, neurogenesis, angiogenesis, pain perception, and mood regulation. Several studies have highlighted the crucial role of the PROK system in the development and maturation of both male and female human reproductive organs. Particularly in men, the PROK system represents a new system useful to clarify some aspects of testicular pathophysiology and provide new potential hypotheses for therapeutic intervention. This narrative review aims to illustrate the state of the art regarding, in particular, the role of PROK2 in male infertility.

## 1. Introduction

The World Health Organization considers infertility as a specific pathology and defines it as the absence of conception after 12/24 months of regular unprotected targeted sexual intercourse [1]. Worldwide, infertility affects about 10–12% of couples. Specifically, male infertility represents about 50% of the causes of infertility in couples [2]. It is estimated that about 5–10% of men of reproductive age have or have had problems in reproduction [3,4]. The diagnosis process is extremely important for defining the etiology, if possible, and consequently to identify therapeutic approaches (pharmacological, surgical, or both) aimed at overcoming the problem [5]. In the last few years, several authors have attempted to identify early and indicative markers capable of providing information on the biological origin of infertility and increasing current knowledge of new potential therapeutic approaches [6,7,8,9]. In this context, the prokineticin system (PROK system) represents a new endogenous system that, playing an important role in the maturation of reproductive organs in humans, is a candidate for an interesting new field of research. 

In the present review, we will briefly illustrate the main recognized causes of male infertility, and the possible involvement of the PROK system. Specifically, we will analyze the most recent knowledge (pre-clinical and human studies) on how the PROK system (in particular, PROK2) can be considered a useful tool for diagnosis and therapy.

Spermatogenesis and testosterone production are regulated by an integrated control system. The hypothalamus, through the pulsatile secretion of gonadotropin-releasing hormone (GnRH), controls the pituitary secretion of follicle-stimulating hormone (FSH) and luteinizing hormone (LH), which in turn stimulate the testicle to produce respectively seminal fluid and testosterone [10,11,12]. The testis has two compartments: the interstitium and the seminiferous tubules [13]. The interstitium contains Leydig cells that produce the male hormone testosterone [13]. The seminiferous tubules and interstitial tissue are separated by a thin connective layer, the lamina propria. The tunica albuginea is the connective tissue coat covering the external surface of the testis. The tubular compartment represents the wider part of the gonad, composed mainly of seminiferous tubules, coiled structures that are 150–250 mm in diameter and can reach more than 80 cm in length. The seminiferous epithelium, which forms the wall thickness of the seminiferous tubule, consists of supporting Sertoli cells and spermatogenic cells in the various stages of maturation: spermatogonia, primary and secondary spermatocytes, and early and late spermatids. Early spermatids are not released into the tubular lumen in physiological conditions, but they complete the maturation process into late spermatids. After the completion of spermiogenesis, late spermatids are released into the tubular lumen as immature sperm. The maturation of sperm cells occurs in the epididymal duct. The seminiferous tubules end in a system of narrow straight ducts called tubuli recti. These straight ducts are devoid of spermatogenic cells and empty into flattened spaces called the rete testis, located in the thickening of the tunica albuginea, called the mediastinum [14,15,16,17,18]. Sperm cells leave the testicles through a complex system that follows the rete testis: they reach the ductuli efferentes, 12–20 ducts with an irregular lumen, which fuse with the head of the epididymis. They cross the caput, corpus, and cauda epididymides, and reach the vas deferens and, subsequently, the seminal vesicles. It should be noted that the sperm cells remain quiescent in the epididymal cauda upon ejaculation [17,18]. Any dysfunction or blockage of spermatogenesis or damage to these structures can lead to impaired fertility. It is not always possible to identify a cause of male infertility. Moreover, various data from both the United States and Europe show that the ejaculate quality is progressively decreasing over the years [3]. In fact, 50 years ago, at least half of men in their 30s had about 100 million spermatozoa per milliliter of seminal fluid; thirty years later, only 20% of males of the same age had the same sperm count. Among the possible causes related to the increase in infertility, certain environmental factors (environmental and workplace pollution) and lifestyle modifications (stress, eating habits, alcohol, smoking) could play a decisive role [19].

The causes of male infertility can be divided into endocrine-related (diseases of the hypothalamus, pituitary gland, or other endocrine diseases), testicular (changes in sperm production), and post-testicular (alterations of the seminal tracts and sexual dysfunctions) [20]. Endocrine-related causes are represented by diseases of the glands that regulate the development and the maintenance of normal spermatogenesis. Hypogonadotropic hypogonadism (HH) is a complex and frequently congenital disease in which the hypothalamus-pituitary stimulation may be affected, and consequently, the testicle can not develop its regular function in the production of male hormones and spermatozoa [21]. Among these, the most frequent condition is represented by congenital HH, which is caused by deficient production, secretion or action of GnRH. When associated with anosmia or hyposmia, congenital HH is known as Kallmann syndrome. This syndrome results from abnormal embryonic migration of GnRH neurons from their origin in the olfactory placode to the forebrain [22]. 

Testicular causes of infertility can be classified into primary and secondary [23]. Among the primary causes of testicular infertility, most are related to congenital, chromosomal, or genetic anomalies. A fairly common cause of infertility linked to chromosomal alterations is the Klinefelter syndrome, characterized by the presence of an extra X chromosome, leading to a 47 XXY karyotype [24]. Alterations due to inflammatory causes (secondary infertility) are generally the consequences of infections that can cause, in the most serious cases, testicular atrophy with azoospermia [25,26]. The post-testicular causes of infertility are mostly due to an obstruction of the excretory ducts [27].

## 2. Prokineticin System

**Figure 1:**
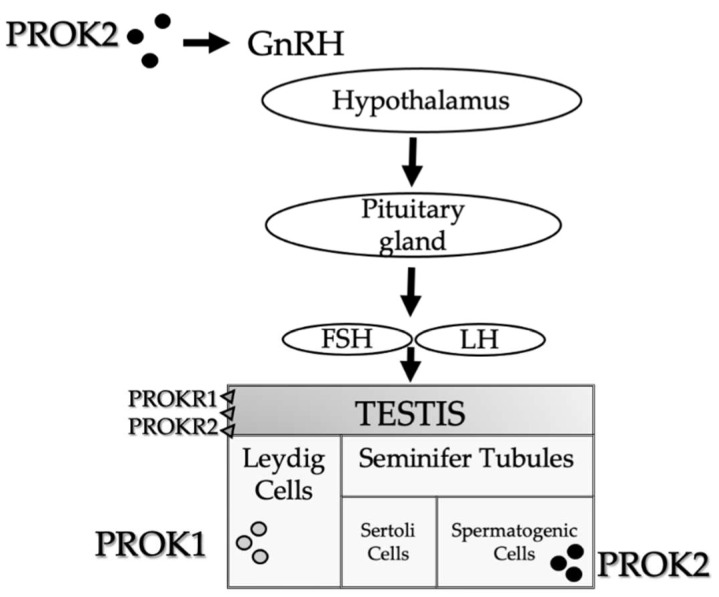
PROKs schematic representation in the male reproductive system. PROK1 is mainly expressed in steroidogenic organs, particularly in the Leydig cells. PROK2 is primarily expressed in the central nervous system, which influences the olfactory bulb development and GnRH neural migration, and in non-steroidogenic cells of the testes. PROK2 expression is limited to the seminiferous tubules in the primary spermatocytes. In the testes, both receptors (PROKR1 and PROKR2) are expressed in endothelial cells in the interstitial tissue.

The prokineticin (PROK) system consists of the prokineticin 1 (PROK1) and prokineticin 2 (PROK2) proteins, identified as the mammalian homologs of two amphibious proteins, the intestinal toxin mamba (MIT-1) and Bv8, respectively. PROKs regulate a wide range of biological functions through the activation of two G-protein receptors: PROKR1 and PROKR2 [28,29]. PROK signaling has been involved in several physiological functions, including gastrointestinal motility [30,31], thus accounting for their family name “prokineticins”, circadian rhythm regulation [32,33,34], neurogenesis [35,36], angiogenesis [37,38,39], pain perception [39,40], mood regulation [41,42,43]. Dysregulation of PROK signaling has been observed in different pathological conditions, such as cancer, ischemia, and neurodegeneration, in which PROK system seems to be a promising therapeutic target (see Figure 1).

Among the numerous biological functions modulated by the PROK system, the role of the development and maturation of the reproductive organs in humans is particularly significant. In general, PROK ligands are considered angiogenic and mitogenic/survival factors, involved in the high rate of endothelial cell turnover and also in the testis [44]. PROK1 is predominantly expressed in steroidogenic organs (ovary, testis, adrenal cortex, placenta), and it has been described to have regulatory properties on the gonads [45,46,47]. In males, PROK1 is abundantly expressed in the testes during embryonal testicular development. Finally, in adult men, PROK1 is expressed in Leydig cells (102),

From the anatomical point of view, PROK2 is predominantly expressed in the central nervous system and non-steroidogenic cells of the testes [34]. Concerning human reproduction, PROK2 plays a major role in olfactory bulb development and GnRH neural migration. In both humans and mice, PROK2 expression is restricted to the seminiferous tubules in the primary spermatocytes [48]. In the testes, both receptors are expressed on endothelial cells in the interstitial tissue [44]. 

## 3. Prokineticin 2 in Male Reproduction: Physiology and Pathology

The focus of the present review is to update the actual knowledge on the role of PROK2 and its receptor PROKR1 on male infertility, considering evidence gathered from pre-clinical and human studies. 

Several methodologies are available for measuring the ligands (PROK1 and PROK2) and the receptors (PROKR1 and PROKR2). Commercial ELISA kits are commonly used for detection in biological fluids (serum, plasma) or in cellular/tissue lysate or supernatant. Moreover, different primary antibodies against the ligands or their receptors could be used for western blot analysis or immunofluorescence technique of tissue samples.

### 3.1. Pre-Clinical Studies

#### 3.1.1. Varicocele

The potential role of PROK2 in experimental varicocele-induced rat testes has been suggested by Tu et al., and demonstrated a significantly increased expression of PROK2 mRNA in the testis compared to the control group. This work was born by evidence of the involvement of PROK2 in endothelial proliferation and in angiogenesis, both processes particularly critical in the testis. The authors demonstrated that the PROK2 system is involved in the hypoxia-induced testicular injury, hypothesizing, on the one hand, a protective role of PROK2, inducing endothelial proliferation, probably in an attempt to protect germ cells from apoptosis. On the other hand, as a chemokine, PROK2 supported a pro-inflammatory environment, favoring male infertility in the experimental model [49].

A more recent study evaluated the mechanisms affecting spermatogenesis in the presence of varicocele. The authors revealed that the varicocele-induced oxidative stress increased the expression of the PROK2, leading to apoptosis of spermatocytes. The same authors demonstrated that in vitro spermatocyte-derived cell line cultured in the presence of H_2_O_2_, to mimic the oxidative stress state of varicocele, overexpressed both PROK2 mRNA and protein, confirming the modulation of the PROK2 system in this oxidative stress-associated pathological condition [50].

Based on these previous studies, a recent experimental work evaluated the mechanism underlying the positive effect of lycopene, a natural extract with antioxidative and anti-inflammatory properties, on hypoxia-induced testicular injury in rats. It was found that lycopene inhibited the PROK2 expression, and consequently the secretion function and spermatogenic function recovered in the testis [51].

#### 3.1.2. Orchitis

It has been proven that the pathogenesis of orchitis mainly includes inflammatory cytokine imbalance, oxidative stress, apoptosis, and the PROK2 pathway. In lipopolysaccharide-induced acute orchitis, administration of methane, an interesting formulation with beneficial properties [52], decreased pro-inflammatory mediators, and over-expressed the anti-inflammatory interleukin IL-10 in the rat testes [53]. In particular, it was found that methane significantly prolonged rat survival, improved the testis condition, alleviated lipopolysaccharide-induced histological changes, and reduced apoptotic cells in the testes [53]. Furthermore, methane significantly increased superoxide dismutase, decreased malondialdehyde, and reduced testicular expression of PROK2 and PROKR1. Therefore, methane exerts therapeutic effects on acute orchitis and might be a new and convenient strategy for the treatment of inflammation-related testicular diseases. [53]. Indeed, methane, the simplest organic compound, was deemed to have little physiological action for decades. However, recently, many basic studies have discovered that methane has several important biological effects that can protect cells and organs from inflammation, oxidant, and apoptosis [52].

Recently, the NLRP3 (NLR family pyrin domain containing 3), the molecular sensor of the NLRP3 inflammasome, has been identified in mouse, human and non-human primates (marmoset and rhesus macaque) testes. Sertoli cells of all species expressed NLRP3, and the expression preceded puberty. In addition, peritubular cells of the adult human testes expressed NLRP3. *NLRP3* and associated genes (*PYCARD*, *CASP1*, *IL1B*) were also found in isolated human testicular peritubular cells and the mouse Sertoli cell line TM4. Due to the involvement of inflammatory events in male infertility, by using a mouse model of male infertility, a group of researchers identified NLRP3 as a novel player in testicular immune regulation because of its expression in the somatic cells of the testis involved in testicular immune surveillance [54]. Activation of the NLRP3 inflammasome in orchitis promotes the secretion and maturation of IL-1β and, thus, decreases male fertility. Su and co-workers developed a uropathogenic *Escherichia coli* (UPEC) rat orchitis model, through which they investigated the NLRP3 inflammatory pathway proteins in testicular macrophages, and in particular, the expression of CaSR (calcium-sensing receptor), responsible for the NLRP3 activation. Interestingly, the authors found that UPEC infection induced large amounts of PROK2 secreted into the cytoplasm to stimulate the activation of CaSR and activate the NLRP3 inflammasome by increasing the level of calcium ions in the cytoplasm of macrophages. This study puts on evidence a regulatory role of the PROK2 in promoting the NLRP3 pathway [55].

**Figure 2:**
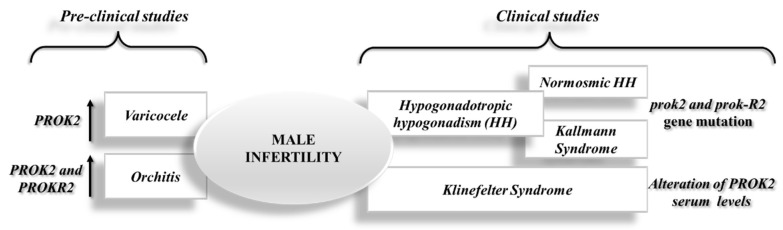
Schematic representation of recent advance on the role of the PROK2 system in male infertility from pre-clinical and clinical studies.

In summary, the most recent pre-clinical studies related to the varicocele and orchitis models disclosed an overexpression of PROK2, a potential new anti-inflammatory therapeutic target for male infertility (see Figure 2).

### 3.2. Clinical Studies

#### 3.2.1. Hypogonadotropic Hypogonadism

As previously said, HH is a disease caused by insufficient stimulation by the luteinizing hormone (LH) and follicle-stimulating hormone (FSH) of otherwise normal functioning gonads. HH can be congenital or acquired, isolated or combined with other secretory defects of the pituitary hormones. The isolated HH can be associated with a normal or altered sense of smell, identifying, respectively, the normosmic HH (nHH) or the Kallmann syndrome. The clinical picture related to HH varies according to the age of onset and can be associated with extra-reproductive clinical manifestations. Kallmann syndrome was originally thought to be caused by mutations in a specific gene located on the X chromosome, KAL1, but it was soon discovered that this genetic defect was present in a minority of patients [56]. Therefore, since the causal event of HH was missing, the classification of “idiopathic” HH (IHH) was used. The observation of family cases with variable modes of inheritance (X-linked or autosomal dominant/recessive) indicated that IHH has a strong genetic component at its base, albeit heterogeneous. In the last decade, contributions from cellular and animal models, together with genetic studies on affected patients, have made it possible to discover new genetic determinants of IHH (both nIHH and Kallmann syndrome) and to better understand their pathophysiology. To date, the identified genes are involved in the development/migration or activation of secreting GnRH neurons, in the synthesis/secretion of GnRH, or in the mediation of the action of GnRH at the pituitary level.

Several studies have shown that PROK2, which maps to chromosome 3p13, and its G protein-coupled receptor, PROKR2, which maps to 20p12.3, play an important role in olfactory and hypothalamic neurogenesis, in both mice and man. Matsumoto and colleagues described the Prokr2-/- knockout mouse in which HH is present with the absence of GnRH-secreting neurons and olfactory bulbs [57]. In 2006, a study by Dodè and colleagues, but also subsequent studies, demonstrated the involvement of variants of these genes in the pathogenesis of IHH also in human patients [58]. These variants interfere in a variable way with the normal activation of the different intracellular signaling pathways elicited by prokineticin. The variants with the greatest phenotypic impact on PROK2 and PROKR2 are those described in homozygosity; however, most of the variants identified, both in KS and nIHH patients, are heterozygous. It is not yet clear whether these heterozygous defects can determine the phenotype through a mechanism of haploinsufficiency or negative dominance on the wild-type receptor. The published data on the possible negative dominance of mutant receptors on wild-type PROKR2 are currently not in agreement. Mutations in prokineticin2 (PROK2) and its cognate receptor PROKR2, a G-protein-coupled receptor, were described in nHH as well [59]. 

A case report described a reversal of HH in a patient with KS carrying a novel homozygous mutation in the PROK-R2 gene (Val274Asp mutation), demonstrating for the first time that reversible KS may be associated with a Prok-R2 mutation [60]. Recently, it has been identified an intron mutation and a new transcription variant in the serum of a Kallmann patient. Considering the close proximity of the mutation site to the intron-exon boundary, the authors speculated that the mutation could impact the regulation of the pre-mRNA splicing isoform of PROK2, favoring the formation of an aberrant PROK2 transcript [61].

#### 3.2.2. Klinefelter Syndrome

Klinefelter syndrome refers to a group of male chromosomal diseases linked to the presence of at least one supernumerary X chromosome compared to the normal male karyotype 46, XY. Although they are often considered as a single nosological entity, this condition should be differentiated from higher-grade aneuploidies (HGAs), in which there is more than one additional X or Y chromosome, which have different clinical, hormonal and metabolic manifestations [62].

Klinefelter syndrome is the most frequent genetic form of male hypogonadism. Klinefelter syndrome symptoms are highly variable and can include late or incomplete puberty, testicular atrophy and low production of testosterone, poor development of facial and body hair, gynecomastia, infertility or reduced fertility, and muscle weakness. Klinefelter patients may also be characterized by other important clinical aspects, including a tendency to develop visceral obesity, dyslipidemia, hypertension, and diabetes mellitus, and hence metabolic syndrome, increasing the cardiovascular risk [63]. In some cases, albeit rarely, there are also delays in language development and reading difficulties (dyslexia). Concerning the last point, our recent study conducted in adolescents affected by Klinefelter syndrome showed reduced brain-derived neurotrophic factor/BDNF serum levels, associated with a decrease in inflammatory markers, disclosing a disrupted immune system and neurotrophins pathways in this pathological condition [64].

Recently, we investigated the serum levels of PROK2 in prepubertal and adult Klinefelter patients, comparing them with healthy subjects. We showed for the first time the presence of PROK2 in the children’s serum but with significant changes in Klinefelter individuals. Indeed, compared with healthy subjects characterized by PROK2 serum elevation during growth, Klinefelter individuals showed constant serum levels during the sexual maturation phase. Indeed, PROK2 serum levels in prepubertal Klinefelter were higher with respect to the young healthy control, but lower during the adult age. Interesting, in adults, Klinefelter PROK2 serum content was independent of testosterone, as in eugonadal, hypogonadal, and hypogonadal individuals under testosterone replacement therapy, PROK2 levels were the same. These data encourage further studies to investigate the role of PROK2 in Klinefelter infertility [65].

## 4. Conclusions

Infertility (especially male infertility) is an increasing problem, especially in developed countries, where genetic, physical, and environmental factors can coexist at the base. Despite the availability of useful tools for diagnosis and therapies to address the problem, in many cases, it is not always possible to recognize a certain cause. In this context, the availability of several new biomarkers to be monitored over time, and the search for new mechanisms on which to hypothesize new therapeutic strategies remains of primary importance. The PROK system, and in particular PROK2, offers an interesting field of study that deserves further investigation to increase knowledge in this important field of medicine (see Figure 2).

## Data Availability

Not applicable.
